# Nonremission and Recurrent Tumor‐Induced Osteomalacia: A Retrospective Study

**DOI:** 10.1002/jbmr.3903

**Published:** 2019-11-15

**Authors:** Xiang Li, Yan Jiang, Li Huo, Huanwen Wu, Yong Liu, Jin Jin, Wei Yu, Wei Lv, Lian Zhou, Yu Xia, Ou Wang, Mei Li, Xiaoping Xing, Yue Chi, Ruizhi Jiajue, Lijia Cui, Xunwu Meng, Weibo Xia

**Affiliations:** ^1^ Department of Endocrinology, Key Laboratory of Endocrinology, NHC Peking Union Medical College Hospital, Chinese Academy of Medical Sciences & Peking Union Medical College Beijing China; ^2^ Department of Nuclear Medicine Peking Union Medical College Hospital, Chinese Academy of Medical Sciences & Peking Union Medical College Beijing China; ^3^ Department of Pathology Peking Union Medical College Hospital, Chinese Academy of Medical Sciences & Peking Union Medical College Beijing China; ^4^ Department of Orthopedic Surgery Peking Union Medical College Hospital, Chinese Academy of Medical Sciences & Peking Union Medical College Beijing China; ^5^ Department of Radiology Peking Union Medical College Hospital, Chinese Academy of Medical Sciences & Peking Union Medical College Beijing China; ^6^ Department of Ear, Nose, and Throat (ENT) Peking Union Medical College Hospital, Chinese Academy of Medical Sciences & Peking Union Medical College Beijing China; ^7^ Department of Stomatology Peking Union Medical College Hospital, Chinese Academy of Medical Sciences & Peking Union Medical College Beijing China; ^8^ Department of Ultrasound Diagnosis Peking Union Medical College Hospital, Chinese Academy of Medical Sciences & Peking Union Medical College Beijing China

**Keywords:** PROGNOSIS, RECURRENT, REFRACTORY, SURGERY, TUMOR‐INDUCED OSTEOMALACIA

## Abstract

Tumor‐induced osteomalacia (TIO) is a rare paraneoplastic syndrome. It is curable by excision of the causative tumor. However, a few cases may persist or relapse after tumor resection. We aimed to investigate the rate of these events and related factors. We retrospectively studied TIO patients treated with surgery in a tertiary hospital. TIO was established based on a pathologic examination or the reversion of hypophosphatemia. Refractory TIO patients consisted of those with nonremission or recurrent hypophosphatemia after surgery. A total of 230 patients were confirmed as having TIO. After primary surgery, 26 (11.3%) cases persisted, and 16 (7.0%) cases recurred. The overall refractory rate was 18.3%. The median time of recurrence was 33 months. Compared with patients in the recovery group, patients in the refractory group were more likely to be female (59.5% versus 41.0%, *p* = .029) and have a lower serum phosphate level (0.44 ± 0.13 versus 0.50 ± 0.11 mmol/L, *p* = .002). The refractory rate was lowest in head/neck tumors (7.5%) and highest in spine tumors (77.8%). Regarding the tissue involved of tumor location, the refractory rate was higher in tumors involving bone than tumors involving soft tissue (32.7% versus 7.0%, *p* < .001). The outcomes of malignant tumors were worse than those of benign tumors (*p* < .001): nonremission rate, 21.4% versus 9.7%; recurrence rate, 28.6% versus 6.5%. In the multivariate regression analysis, female sex, spine tumors, bone tissue‐involved tumors, malignancy, and low preoperation serum phosphorus levels were identified as risk factors for refractory outcomes. High preoperative fibroblast growth factor 23 (FGF23) levels were also associated with refractory after adjusting for involving tissue and tumor malignancy. In summary, we are the first to report the rate and clinical characteristics of refractory TIO in a large cohort. For patients with multiple risk factors, especially spine tumors, clinical practitioners should be aware of a poor surgical prognosis. © 2019 The Authors. *Journal of Bone and Mineral Research* published by American Society for Bone and Mineral Research.

## Introduction

Tumor‐induced osteomalacia (TIO) is the most prevalent form of acquired hypophosphatemic osteomalacia.^1^ In nature, TIO is a paraneoplastic syndrome; the majority of cases are caused by tumors with excess production and secretion of fibroblast growth factor 23 (FGF23), the majority of which are classified as a phosphaturic mesenchymal tumor (PMT) or a PMT, mixed connective tissue variant (PMTMCT).^2^ FGF23 plays a key role in the regulation of phosphorus homeostasis. Its classic effects are inhibition of the expression of type 2a and 2c sodium‐phosphorus cotransporters on proximal renal tubules, which results in reduced phosphorus reabsorption and hypophosphatemia, and inhibition of the production of 1,25‐dihydroxyvitamin D and parathyroid hormone (PTH).^3^ Most clinical symptoms of TIO are the consequences of chronic hypophosphatemia. Almost every patient with TIO seeking treatment suffers from bone pain, which usually starts in weight‐bearing bones, as well as muscle weakness and impaired mobility, and fragility fractures are common.

In such circumstances, localization and resection of the causative tumor are of vital importance. Imaging techniques for locating tumors have been highly developed in recent years and include functioning imaging techniques, such as technetium‐99m octreotide scintigraphy (^99^Tc^m^‐OCT), anatomical imaging techniques, such as MRI, and the combination of functioning and anatomical imaging techniques, such as ^68^gallium‐DOTATATE‐positron emission tomography/computed tomography (^68^Ga‐DOTATATE‐PET/CT).

Once the tumor is located, tumor excision is the cornerstone of treatment. Although hypophosphatemia and its related symptoms recover after tumor excision in most patients, there are sporadic reports of nonremission or recurrent cases.^4^, ^5^ These patients need multiple resection surgeries, which may mean an increased risk of surgical damage and multiple risks. Recognition of these refractory cases and understanding the prognosis of surgery treatment are crucial for clinical practice. However, to date, data on nonremission and recurrent TIO cases are scarcely reported. In this study, we aimed to investigate the incidence rate of refractory cases and related risk factors in a large TIO cohort.

## Patients and Methods

### Patients

Patients who were suspected of having TIO and underwent tumor excision surgery at Peking Union Medical College Hospital (PUMCH) between January 1, 2004, and January 31, 2018, were retrospectively studied. The period between the last surgery and data collection was at least 1 year. A diagnosis of TIO was confirmed if the tumor was a PMT/PMTMCT according to the pathological examination or if the patient's serum phosphorus level returned to normal after surgery. Then, these TIO patients were divided into three groups: the nonremission group, which included patients whose postoperative serum phosphorus levels remained low; the recurrent group, which included patients whose serum phosphorus levels returned to normal for at least 1 month but hypophosphatemia and symptoms recurred; and the recovery group, which included patients whose serum phosphorus levels increased and remained higher than the lower level of the reference range until the last visit. A patient was considered to have a malignant tumor when at least one of the patient's tumors was diagnosed as a malignant tumor by our pathologists according to the criteria described in Histopathology or if the patient had distant metastasis. All patients included must have been followed for at least 1 month after surgery. The reasons for nonremission or recurrence were divided into imperfect resection, distant metastasis, multiple lesions, or distant metastasis combined with multiple lesions. For patients who did not have multiple lesions or distant metastasis, although “complete resections” were conducted, we still considered that there were imperfect resections if no other tumors were located when the disease persisted or recurred after the primary surgery.

### Tumor locating

Patients with suspected TIO first received a physical examination and functional imaging, including ^99^Tc^m^‐OCT or ^68^Ga‐DOTATATE‐PET/CT. If suspicious lesions were found, anatomical imaging, including ultrasonography, CT, or MRI, was performed at specific sites. ^99^Tc^m^‐OCT and ^68^Ga‐DOTATATE‐PET/CT were performed by the Department of Nuclear Medicine of PUMCH. Ultrasonography was performed by the Department of Ultrasound of PUMCH, and CT and MRI were performed by the Department of Radiology of PUMCH.

### Measurement of FGF23

Serum intact FGF23 (iFGF23) levels were assessed with enzyme‐linked immunosorbent assay (ELISA) kits (KAINOS Laboratories, Tokyo, Japan). The detailed procedure has been described.^6^ This is a two‐site ELISA kit for the measurement of FGF23 in the serum. The kit has a quantification range of 3 to 800 pg/mL. Samples with FGF23 levels exceeding this range were diluted 10 times for further measurement.

### Histopathology

The World Health Organization defined PMT in the 2013 Classification of Tumors of Soft Tissue and Bone as “morphologically distinctive neoplasms that produce tumor‐induced osteomalacia in most affected patients, usually through production of fibroblast growth factor 23.” Usually, these tumors are characterized by their low cellularity, myxoid change, bland spindled cells, distinctive “grungy” calcified matrix, fat, hemangiopericytoma (HPC)‐like vessels, microcysts, hemorrhage, osteoclasts, and an incomplete rim of membranous ossification.^7^ The diagnosis of malignant tumors was based on distant metastasis or the histopathological findings of “frankly sarcomatous features” (a high mitotic rate, prominent atypia and pleomorphism, and necrosis), high cellularity, a high Ki‐67 index (a proliferation marker), a high nuclear grade, and a high degree of invasion.^7^ We must stress here that there is no consensual histopathological criteria for nonmetastatic malignant PMTs. The Ki‐67 labeling index was calculated in approximately 1000 cells. Five microscopic areas (magnification ×400) were selected for counting, including the most densely labeled areas. All pathological results were reconfirmed by a senior pathologist during the review.

### Statistical analysis

Quantitative data are presented as the mean ± standard deviation (SD) or the median (interquartile range [IQR]), and differences between two groups were compared with Student's *t* test and the Mann‐Whitney *U* test for normally distributed variables and non‐normally distributed variables, respectively. Categorical data are presented as frequencies and percentages (%) and were analyzed using the chi‐square test and Fisher's exact test. Logistic regression analysis was used to seek factors related to treatment outcomes. Factors having significant correlations with outcomes in the univariable analysis were included in the multivariable analysis with the likely ratio forward stepwise method. Concerning the relatively small absolute number of refractory cases, we used three regression models that contained five, six, and seven variables to corroborate our results. Although FGF23 levels were available in only 21 refractory cases and 65 recovery cases, we still analyzed the influence of preoperative FGF23 levels on outcomes in these patients because it is a factor reflecting tumor function directly. Available FGF23 levels were divided into four stratifications according to the quartile and used in the univariate and multivariate regression analyses. Because the sample size of the refractory group was small in this multivariate analysis, we adjusted only for the two most influential factors on the basis of the previous regression analysis, which were the involved tissue and malignancy. Associations between two normally distributed continuous variables were analyzed by Pearson's correlation coefficient, and associations between categorical variables and continuous variables or associations between non‐normally distributed variables were analyzed by the Spearman rank correlation coefficient. Receiver operating characteristic (ROC) curves were generated with the Wilson/Brown method on GraphPad Prism version 8.0.2 (GraphPad Software, Inc., La Jolla, CA, USA). The area under the ROC curve (AUC) was calculated and compared with 0.5. Youden's index, defined as (specificity + sensitivity – 1), was utilized to select the optimal cutoff point.

A *p* value <.05 was considered statistically significant. All statistical analyses were performed with SPSS Statistics version 23 (IBM Corp., Armonk, NY, USA) and GraphPad Prism for Windows version 8.0.2.

## Results

**Figure 1:**
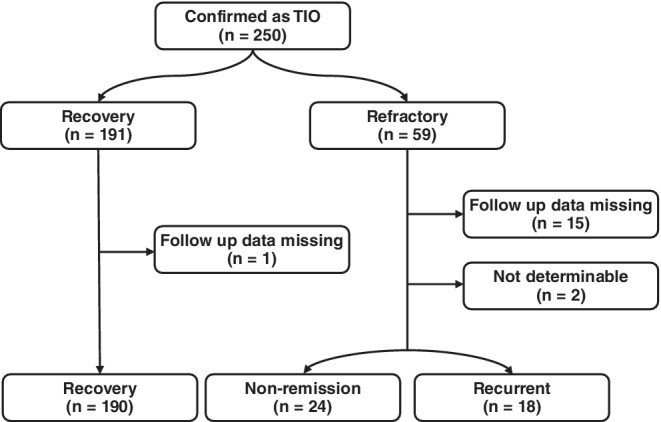
Flowchart of enrollment.

**Figure 2:**
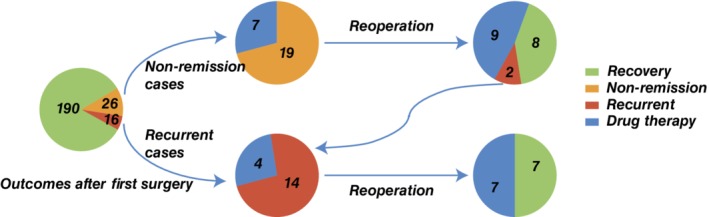
General outcomes of 230 TIO patients. After primary surgery, 26 patients did not recover. Among them, 19 received multiple surgeries, with nine patients showing persistent hypophosphatemia. Among the 10 patients who recovered, two experienced recurrence and were enrolled in the recurrent group. Therefore, 14 recurrent patients received multiple surgeries, and seven patients recovered.

The enrollment procedure is presented in Fig. 1. A total of 249 patients with suspected TIO were treated with tumor resections and confirmed as having TIO. Among them, 17 patients were excluded because of a lack of follow‐up data, and two patients were excluded because their postoperative serum phosphorus levels fluctuated above and below the lower reference range and their outcomes were not determinable. Therefore, 230 patients with TIO were enrolled in this study. The overall outcomes are shown in Fig. 2. After the first surgery, there were 26 patients with persistent TIO and 16 patients with recurrent TIO. Nineteen patients with persistent TIO received multiple surgeries (eight patients recovered, nine patients persisted, and two patients experienced recurrence and were classified into the recurrent group). Therefore, there were 24 patients in persistent group and 18 patients in recurrent group. On the other hand, 14 of all patients who experienced recurrence received multiple surgeries, and one‐half of them recovered. It took a median of 5.5 days (range, 1 to 18 days) for the postoperative serum phosphate level to return to a normal range. The median time to recurrence was 33 months. The reasons for nonremission and recurrence are summarized in Supplemental Table 1. Except for six patients with multiple lesions or distant metastasis, imperfect resection was still the major reason. At the time this manuscript was written, 81.7% (188/230) of TIO patients recovered after a single surgery, whereas 45.5% (15/33) of patients who underwent multiple surgeries recovered, suggesting that the recovery rate of multiple surgeries was significantly lower than primary surgery (*p* < .001, data not shown); the overall recovery rate was 88.3% (205/230).

### General characteristics of patients with refractory TIO

The general characteristics of refractory cases are shown in Table 1. After primary surgery, the incidence of nonremission and recurrent TIO were 10.4% (24/230) and 7.8% (18/230), respectively, with an overall refractory rate of 18.3% (42/230). A total of 59.5% (25/42) of the patients were female. The majority of refractory patients were adult‐onset, with a mean age of 34 years and underwent tumor excision surgery twice (range, 1 to 7 times), and the mean duration between initial complaints and the first surgery was 4 years (range, 1 to 27 years). All patients suffered from bone pain and gait abnormalities. For laboratory tests, their mean serum phosphorus levels were 0.44 mmol/L (range, 0.19 to 0.68 mmol/L), with normal serum calcium levels and elevated alkaline phosphatase (ALP) levels. More than one‐half of the patients had elevated PTH levels (24/42, 57.1%). They all showed excessive urine phosphorus excretion, which conformed to the pathophysiology of TIO. The median follow‐up duration was 4 years (range, 0.2 to 29 years).

**Table 1:** Clinical Characteristics of 42 Refractory Patients with TIO

Characteristic	Value	Range	Reference range
Incidence in cohort, % (*n*/*n*)	18.3 (42/230)		
Gender, female, *n* (%)	25 (59.5)		
Onset age (years), mean ± SD	34.6 ± 12.6	14.0–62.0	
Number of operations, median (IQR)	2 (1.75, 2.00)	1–7	
Duration (years), median (IQR)	4.0 (2.8, 7.0)	1.0–27.0	
Bone pain, *n* (%)	42 (100)		
Gait abnormalities, *n* (%)	42 (100)		
Bone deformity, *n* (%)	15 (35.7)		
Serum phosphate (mmol/L), mean ± SD	0.44 ± 0.13	0.19–0.68	0.81–1.45
Serum calcium (mmol/L), mean ± SD	2.32 ± 0.14	2.08–2.63	2.13–2.70
ALP (U/L), median (IQR)	216.0 (144.3, 293.8)	68.0–642.1	F: 35–100; M: 45–125
PTH (pg/mL), median (IQR)^a^	95.9 (55.8, 140.0)	20.1–560.0	12.0–68.0
25OHD (ng/mL), median (IQR)^a^^,^ b	21.2 (12.5, 24.3)	5.4–88.0	
Creatinine (μmol/L), mean ± SD^a^	58.27 ± 15.46	32‐92	F: 45–84; M: 59–104
24 Hour urine phosphorus (mmol), median (IQR)^a^	23.03 (13.4, 47.7)	6.60–90.00	
TMP/GFR (mmol/L), mean ± SD^a^	0.34 ± 0.13	0.18‐0.64	
Follow‐up duration (years), median (IQR)	4.0 (2.0, 8.3)	0.2–29.0	

SD = standard deviation; IQR = interquartile range; ALP = alkaline phosphatase; PTH = parathyroid hormone; 25OHD = 25‐hydroxyvitamin D; TMP/GFR = tubular maximum reabsorption of phosphate/glomerular filtration rate.

a For PTH, 25OHD, creatinine, 24‐hour urine phosphorus, and TMP/GFR, data were collected from 39, 30, 33, 35, and 21 cases, respectively.

b For 25OHD, 1 ng/mL = 2.5 nmol/L.

### Different characteristics between the refractory and recovery groups

The comparison of clinical characteristics between the two groups is presented in Table 2. Compared to the recovery group, the refractory group was younger (34.6 ± 12.6 versus 38.9 ± 12.0 years, *p* = .041), the female/male ratio was higher (25/17 versus 77/111, *p* = .029), and the serum phosphorus levels were lower (0.44 ± 0.13 versus 0.50 ± 0.11 mmol/L, *p* = .002). ALP levels were lower in the refractory group (216.0 [144.3, 293.8] versus 274.0 [195.3, 366.5] U/L, *p* = .004), whereas β‐C‐terminal telopeptide of type I collagen (β‐CTx) levels were not significantly different (0.676 [0.500, 0.909] versus 0.600 [0.400, 0.843] ng/mL, *p* = .584). The courses of disease before the first surgery were similar (4.0 [2.8, 7.0] versus 4.0 [2.0, 6.0] years, *p* = .318). According to available preoperation FGF23 data (21 patients in the refractory group and 65 patients in the recovery group), the FGF23 levels were dramatically higher in refractory cases than in recovery cases (1342.07 [386.08, 2030.73] versus 323.75 [190.33, 541.72] pg/mL, *p* < .001). Postoperative FGF23 levels were also available for some patients. Patients with postoperative FGF23 levels below the lower limit of the measurable range were more common in the recovery group than in the refractory group (1/14, 7.1% versus 26/54, 48.1%; *p* = .005). The proportion of causative tumors with Ki‐67 indexes ≤1 in 31 refractory patients and 49 recovery patients was similar (9/31, 29.0% versus 15/49, 30.6%; *p* = .881).

**Table 2:** Comparison of Clinical Characteristics between Refractory and Recovery Cases

Characteristic	Refractory	Recovery	*p*
Onset age (years), mean ± SD	34.6 ± 12.6	38.9 ± 12.0	**.041**
Gender, F:M (*n*/*n*)	25:17	77:111	**.029**
Premenopausal rate in female, %	72.0	72.7	.994
Duration (years), median (IQR)	4.0 (2.8, 7.0)	4.0 (2.0, 6.0)	.523
Serum phosphate (mmol/L), mean ± SD	0.44 ± 0.13	0.50 ± 0.11	**.002**
ALP (U/L), median (IQR)	216.0 (144.3, 293.8)	274.0 (195.3, 366.5)	**.004**
β‐CTx (ng/mL), median (IQR)	0.676 (0.500, 0.909)	0.600 (0.400, 0.843)	.584
FGF23 before surgery (pg/mL), median (IQR)^a^	1342.07 (386.08, 2030.73)	323.75 (190.33, 541.72)	**<.001**
Creatinine (μmol/L), mean ± SD^b^	58.27 ± 15.46	57.44 ± 13.86	.756
Ki‐67 ≤ 1 (%)^c^	29.0	30.6	.881

Bold values are significant.

SD = standard deviation; IQR = interquartile range; ALP = alkaline phosphatase; β‐CTx = C‐terminal cross‐linked telopeptide of type I collagen; FGF23 = fibroblast growth factor 23.

a Preoperative FGF23 levels were available in 21 refractory patients and 65 recovery patients.

b Creatinine levels were available in 33 refractory patients and 170 recovery patients.

c Ki‐67 indexes of primary tumors were available in 31 refractory patients and 49 recovery patients.

**Figure 3:**
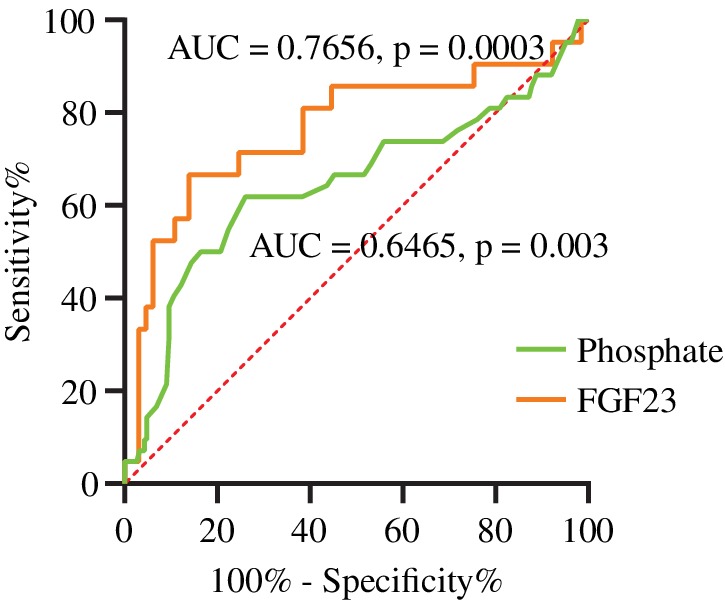
ROC curves of preoperative phosphate and FGF23 levels to predict outcomes. The green curve indicates the phosphate level, and the orange curve indicates the FGF23 level. The AUCs were 0.6465 for phosphate and 0.7656 for FGF23, both significantly greater than 0.5. ROC = receiver operating characteristic; AUC = area under the curve.

To define the optimal cutoff points of preoperative phosphate and FGF23 levels to predict refractory outcomes, we established ROC curves and calculated Youden's index (Fig. 3). For preoperative phosphate, the AUC was 0.6465 (95% confidential interval [CI], 0.5397 to 0.7534; *p* = .003), and the optimal cutoff point to predict refractory outcomes was <0.445 mmol/L (sensitivity 61.9%, specificity 73.9%, Youden's index 0.3584). For preoperative FGF23, the AUC was 0.7656 (95% CI, 0.6298 to 0.9014; *p* = .0003), and the optimal cutoff point to predict refractory outcomes was >709.8 pg/mL (sensitivity 66.67%, specificity 86.15%, Youden's index 0.5284). The difference between the two curves could not be compared because the FGF23 data were incomplete. The full sensitivity and specificity results of phosphate and FGF23 levels are shown in Supplemental Tables 2 and 3, respectively.

In addition, we divided serum phosphate levels into seven intervals to determine their distribution and the refractory rate in each interval (Supplemental Fig. 1). We found that serum phosphate levels in the majority of refractory cases were approximately 0.31 to 0.4 mmol/L, whereas those in recovery cases were mainly within the range of 0.41 to 0.6 mmol/L. The refractory rate in each interval generally decreased as the phosphate levels increased.

### Different distributions of tumors between the refractory and recovery groups

**Figure 4:**
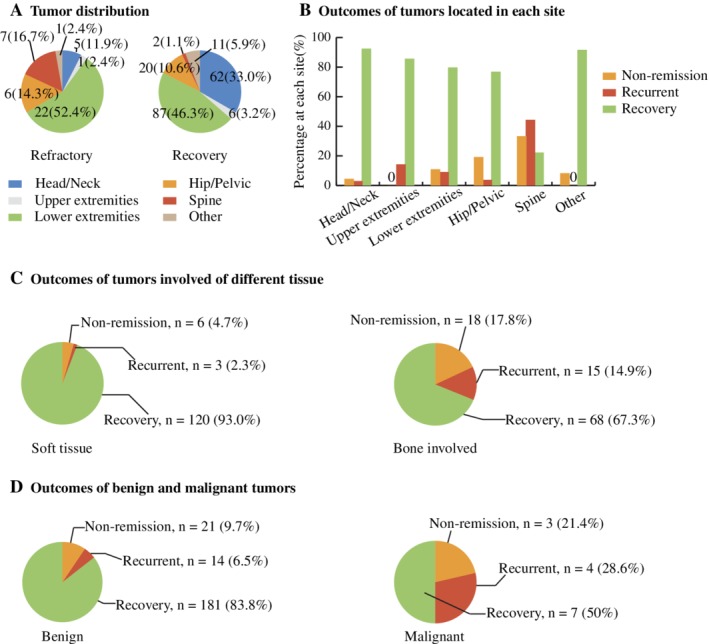
Outcomes of tumors with different characteristics. (*A*) Tumor locations of refractory cases and recovery cases. The value in each sector indicates the number of tumors (percentage in each group) of each location. (*B*) Tumor outcomes at each location. There was no nonremission case in the upper extremities and no recurrent case in the “other locations group.” (*C*) Outcomes of soft tissue tumors and bone tissue–involved tumors. (*D*) Outcomes of benign and malignant tumors.

Next we summarized the site distribution of causative tumors (Fig. 4 *A*). For tumors with metastasis, only the location of the primary tumor was calculated. Approximately one‐half of the tumors were located in the lower extremities either in the refractory group or in the recovery group. Tumors in the head/neck were less common (5/42, 11.9% versus 62/188, 33.0%, *p* < .001) whereas tumors in the spine were more common (7/42, 16.7% versus 2/188, 1.1%, *p* < .001) in the refractory group than in the recovery group. The proportions of hip/pelvic, upper extremity, lower extremity, and other tumors were similar between the two groups. The refractory rate at each site was 7.5% (5/67) in the neck/head, 14.3% (1/7) in the upper extremities, 20.2% (22/109) in the lower extremities, 23.1% (6/26) in the hip/pelvis, 77.8% (7/9) in the spine, and 8.3% (1/12) in other sites (Fig. 4 *B*). Tumors located in the spine showed a significantly higher refractory rate than those located in the head/neck, upper extremities and other sties (*p* < .001). Although the refractory rates of tumors located in the upper extremities and hip/pelvis seemed lower than those of the spine, there was no significant difference, probably because of the relatively small sample size.

TIO tumors were classified into soft tissue tumors or bone tissue–involved tumors, which included tumors at bone sites and tumors involving soft tissue and bone tissue simultaneously. There were 129 soft tissue tumors (129/230, 56.1%) and 101 bone tissue‐involved tumors (101/230, 43.9%). Outcomes were significantly different between these two tumor types (*p* < .001): for soft tissue tumors, 93.0% (120/129) recovered, 2.3% (3/129) relapsed, and 4.7% (6/129) persisted; for bone tissue‐involved tumors, 67.3% (68/101) recovered, 14.9% (15/101) relapsed, and 17.8% (18/101) persisted (Fig. 4 *C*).

### Histological characteristics of tumors in the refractory group

Most tumors were characterized as PMT or PMTMCT (222/230, 96.5%). Other tumors included odontogenic fibromas, hemangiomas, and so on. A portion of these tumors may be characterized as PMT mixed epithelial and connective tissue type, which is a new variant of PMT recently proposed.^8^ In addition, 6.1% (14/230) of all TIO tumors were malignant (in this study, we defined a tumor as a malignant tumor if distant metastasis occurred or if its histopathology suggested malignancy). Among them, four malignant tumors were multifocal, and all four tumors came from the lower extremities. The outcomes of malignant tumors were significantly worse than those of benign tumors (*p* < .001, Fig. 4 *D*): nonremission rate, 21.4% (3/14) versus 9.7% (21/216); recurrence rate, 28.6% (4/14) versus 6.5% (14/216); recovery rate, 50.0% (7/14) versus 83.8% (181/216).

### Risk factors for refractory TIO

Based on the above results, we performed univariate logistic analyses first (Table 3). Outcomes were divided into recovery and refractoriness. Age, sex, tumor location, involved tissue, malignancy, and preoperative serum phosphate and ALP levels were analyzed. Older onset age, male sex, soft tissue tumor, benign tumor, higher preoperation serum phosphorus levels and higher ALP levels were identified as protective factors for recovery. For tumor location using the head/neck as a reference, those in the lower extremities, hip/pelvis, and spine showed a higher risk of refractoriness.

**Table 3:** Risk Factors Associated with Refractory Cases

	Univariate	Multivariate		
Factor	OR (95% CI)	OR (95% CI)^a^	OR (95% CI)^b^	OR (95% CI)^c^
Onset age, OR per increase 1 year	0.97 (0.94–1.00)^d^	Excluded from equation		
Gender				
Male	1	1	1	1
Female	2.12 (1.07–4.19)^d^	2.83 (1.25–6.37)^d^	3.54 (1.48–8.50)^e^	3.54 (1.48–8.50)^e^
Tumor location				
Head/neck	1	1	1	1
Upper extremities	2.07 (0.21–20.71)	3.95 (0.30–52.04)	2.43 (0.15–39.36)	2.43 (0.15–39.36)
Lower extremities	3.14 (1.13–8.73)^d^	2.68 (0.90–8.02)	2.77 (0.90–8.54)	2.77 (0.90–8.54)
Hip/pelvic	3.72 (1.03–13.51)^d^	3.44 (0.86–13.75)	3.21 (0.76–13.57)	3.21 (0.76–13.57)
Spine	43.40 (7.06–266.93)^f^	40.81 (6.02–276.56)^f^	61.40 (8.10–465.40)^f^	61.40 (8.10–465.40)^f^
Other	1.13 (0.12–10.60)	1.65 (0.15–18.15)	1.81 (0.16–20.08)	1.81 (0.16–20.08)
Involved tissue				
Soft tissue	1	1	1	1
Bone involved	6.47 (2.92–14.33)^f^	6.88 (2.78–16.99)^f^	7.35 (2.89–18.69)^f^	7.35 (2.89–18.69)^f^
Malignancy				
Benign	1	1	1	1
Malignant	5.17 (1.71–15.67)^e^	4.58 (1.33–15.75)^d^	4.18 (1.14–15.28)^d^	4.18 (1.14–15.28)^d^
Preoperative serum phosphorus, per increase 0.1 mmol/L	0.63 (0.46–0.86)		0.54 (0.37–0.79)^e^	0.54 (0.37–0.79)^d^
ALP before operation, per increase 10 U/L	0.96 (0.94–0.99)^e^	Excluded from equation		

OR = odds ratio; CI = confidential interval; ALP = alkaline phosphatase.

a Model 1: Including onset age, gender, tumor location, involved tissue and malignancy.

b Model 2: Model 1 + preoperative serum phosphorus.

c Model 3: Model 1 + preoperative serum phosphorus + ALP.

d *p* < .05.

e *p* < .01.

f *p* < .001.

Then, the variates significantly associated with outcomes in the univariate analyses (*p* < .05) were included in the multivariate analyses using the likely ratio forward stepwise method (Table 3). Considering that the relatively small sample size would affect the stability of the equations, we used three models consisting of five to seven variates to analyze the risk of refractoriness, and the results were comparable. Onset age, sex, tumor location, involved tissue, and malignancy were included in the analysis in model 1, serum phosphate was added to model 2, and serum phosphate and ALP levels were added to model 3. With the likely ratio forward stepwise method, age was excluded from all models, and the ALP level was excluded from model 3 during the analyzes. Hence, model 2 was the same as model 3. Female sex, bone tissue–involved tumors, and malignant tumors showed reliable features in predicting refractory cases (model 2: OR = 3.54, 7.35, and 4.18; *p* < .01, *p* < .001, and *p* < .05, respectively). Compared with head/neck tumors, the refractory risk was dramatically higher for tumors located in the spine (OR = 40.81 and 61.40 for models 1 and 2, respectively; *p* < .001). In addition, each 0.1 mmol/L increase in preoperative serum phosphorus levels (range, 0.19 to 0.68 mmol/L) reduced the risk of refractoriness by more than 40% (*p* < .01). When we excluded tumor malignancy from our model, the results were similar (Supplemental Table 4). The only new finding was that tumors located in the lower extremities also showed a higher risk than head/neck tumors. This phenomenon could be incidental because of the relatively small number of malignant tumors, and there was no tumor located in the head/neck.

We found that age was excluded from all multivariable models; therefore, the correlations between age and other variables were analyzed (Supplemental Table 5). The results revealed that age was negatively associated with bone tissue–involved tumors, tumor malignancy, and serum ALP levels (correlation coefficient = −0.167, −0.252, and − 0.242; *p* = .011, *p* <.001, and *p* <.001; respectively) and positively associated with female sex and serum phosphate levels (correlation coefficient = 0.148 and 0.175, *p* = .025 and *p* = .008, respectively). These results revealed that age did not influence the outcome independently.

Considering that the preoperative FGF23 level may be an important factor associated with outcomes, we also performed regression analyses including FGF23 in 86 subjects with FGF23 data (Supplemental Table 6). Available FGF23 levels were categorized into four stratifications (quartile 1, <211.47 pg/mL; quartile 2, 211.47 to 383.77 pg/mL; quartile 3, 383.78 to 819.37 pg/mL; and quartile 4, >819.37 pg/mL). The increase per quartile increased the risk of developing refractory outcomes by 2.5‐fold in the univariate analysis (OR = 2.484, *p* = .001). After adjusting for involved tissue and malignancy, a quartile increase in FGF23 still increased the risk of developing refractory outcomes by threefold (OR = 3.278, *p* = .001).

### Subgroup analysis

Because the median time to recurrence was 33 months in this cohort, we screened the recovery group again and included only patients who received surgeries 33 months prior. All of the above analyzes were performed again, and the results were similar (data not shown). We also compared the characteristics between the nonremission group and the recurrent group and found no significant difference (Supplemental Table 7), except for preoperative FGF23 levels (1789.41 [1229.86, 2044.63] versus 374.67 [109.57, 387.48], *p* < .001).

## Discussion

TIO is a rare disease. Its incidence or prevalence is still unclear, and only one Japanese study reported an estimated annual incidence of 117 cases nationally.^1^ As the most prevalent cause of acquired hypophosphatemic osteomalacia, TIO is not as common as other common FGF23‐related hypophosphatemic diseases, such as X‐linked hypophosphatemia, which requires long‐term drug therapy and is curable by tumor resection. Hence, comprehending the prognosis of surgical treatment and finding predictors are of great importance. Our study first addressed this issue in TIO by studying the largest TIO patient cohort with the outcomes of surgical treatment. We found that the prognosis of tumor resection was promising, but patients with some specific characteristics were at high risk of persistent or recurrent TIO.

Most patients recovered after primary surgery, and reoperations still showed effectiveness in approximately one‐half of refractory cases. The overall recovery rate was 88.4% in our cohort. Previously reported recovery rates in other studies have varied from 72.7% to 100%.^4^, ^5^, ^9^, ^10^, ^11^, ^12^ Small sample sizes, gaps in medical resources, focuses on different tumor locations, and different follow‐up periods of these studies all led to discrepancies. The result of our cohort may provide convincing evidence for this predicament. We found that the disease was equally prevalent in women and men, which is consistent with previous reports,^1^, ^4^, ^11^ but our results suggest that the refractory risk in women is twofold to threefold higher than that in men. To our knowledge, this finding is difficult to explain. This could be because of accidental statistical differences or some unknown latent mechanisms, similar to that reported in thyroid cancer in men.^13^ Onset age also affects patient outcomes. However, the influence was not statistically significant in the multivariable analyses. A possible explanation for this finding is that a younger onset age is associated with the presence of malignant or bone tissue–involved tumors, similar to our correlation analyses, which consequently lead to worse outcomes.

Among each tumor location, head/neck tumors exerted the highest recovery rate, whereas spine tumors were most likely to be refractory. More than 70 cases in the head/neck have been reported in addition to the cases reported from our institute, and only 10 cases among them were refractory.^4^, ^11^, ^12^, ^14^, ^15^, ^16^ Although this rate is higher than ours, it is plausible that TIO tumors in the head/neck have a favorable outcome. It does not make sense to compare the delicate differences between our cases and those reported in various institutes because other cases are so scattered. TIO tumors in the spine are even more rare, with only 18 cases reported in the literature before the current study.^17^, ^18^, ^19^ Our nine spine cases enriched the current limited experience of this peculiar type of TIO. Despite the small sample size, we observed unusually poor outcomes of these cases, with more than two‐thirds of tumors being persistent or recurrent. This finding implies that the difficulties associated with complete resections of spine TIO tumors and occult residual tumors may be more common than we expected. Spine surgeons need to determine the excision extension prudently, and chemotherapy and radiotherapy may be considered in the future. Even if the biochemical findings normalize after surgery, intense monitoring is required for these patients.

Regardless of nonremission or recurrence, these refractory cases represent the failure to completely excise TIO tumors. The tumor itself and difficulty of the operation both influence the outcome. For tumors, invasive activity has a substantial effect on prognosis, similar to all other tumors. Our finding that malignant cases were more likely to relapse verified this view in TIO tumors. In addition, the secretory function of TIO tumors, which is their unique feature, seems to affect the outcomes of TIO. Serum FGF23 levels before the operation were found to be significantly higher in some refractory cases than in recovery cases. Our simplified regression model also suggested preoperative FGF23 levels as a risk factor for refractory outcomes. Furthermore, the preoperative FGF23 level could be a barely satisfactory predictor of refractory outcomes, as shown in Fig. 3. Future studies with complete FGF23 data are necessary to confirm this result. However, we should note that inconsistent results about FGF23 could be obtained by different measuring methods. As FGF23 data were not available in all patients, we used fasting serum phosphorus levels as a surrogate marker of the secretory function of tumors. Lower phosphorus levels were identified as a risk factor for refractoriness even after adjusting for other factors in the multivariable analysis, and the preoperative phosphate levels also had significant value in predicting outcomes. However, the AUC of phosphate seems to be lower than that of FGF23. The statistical comparison of these two AUCs was meaningless because the FGF23 data were incomplete. Above all, the best cutoff values of phosphate and FGF23 presented limited sensitivity and specificity, which probably indicated that tumor location or surgical procedure were more important factors for outcomes. Due to its easy accessibility, serum phosphorus can be a preliminary crude marker for prognosis judgment. However, as the exact size of each tumor was unavailable, we could not determine whether the higher secretory function resulted from higher‐functioning tumor cells or whether it was a matter of only a larger tumor size. The difficulty of surgery also plays a crucial role in the prognosis of TIO. The poor prognosis of spine tumors is a case in point. In addition, the better outcomes associated with soft tissue tumors rather than bone tissue–involved tumors also stress the importance of surgical difficulty. For example, Sun and colleagues^9^ performed complete resection for soft tissue tumors and extensive tumor curettage for bone tissue‐involved tumors and found that tumor resection was the preferred option for extremity TIO tumors compared with curettage surgery. Bone tissue‐involved tumors actually indicate both stronger invasive ability and a more difficult operation. More extensive excision should be considered for these patients, and an experienced surgeon is important for predicting disease outcomes.

Because some tumors in specific locations (such as the spine) are difficult to completely remove, the surgeon's skill and experience are of vital importance for a final cure. TIO tumors are distributed throughout the whole body; for this reason, many surgeons from different departments are involved in the operation, and their expertise can vary. We must realize that different expertise would influence patient outcomes. This is an inevitable limitation of our study. However, all surgeons involved were experienced doctors in TIO treatment, and they followed standard operation procedures to minimalize this discrepancy.

Our study has several other limitations. First, the number of spine cases was small. Although we reported most TIO cases described so far, spine cases are still rare. More cases are necessary for a convincing result. A portion of our patients also lacked FGF23 data. Although we used fasting serum phosphorus levels as a surrogate marker of the secretory function of tumors and found that it was a barely satisfactory predictor for refractory outcomes, FGF23 is the direct product of TIO tumors, and its level should be a better marker of the secretory function than that of phosphorus. Our results indicate that a higher FGF‐23 level may be a promising predictor for refractory outcomes. Further studies are urgently needed to find a more reliable cutoff point for FGF23. Third, because there are no consensual histopathological criteria for malignant PMTs, the diagnoses of malignant PMTs without distant metastasis in our study were partially subjective, and the results related to tumor malignancy should be interpreted with caution. However, all histopathological results were reconfirmed by a senior pathologist experienced in the histopathology of PMTs, and we repeated our regression analyses after excluding tumor malignancy as a variable and found similar results. In addition, we observed only a phenomenon, but the mechanism of refractoriness, especially in pathology studies, is still absent. Here, we must stress again that our results revealed only that patients with specific characteristics were more likely to persist or recur in clinical practice. These outcomes were influenced by both the nature of the tumor itself and the diagnosis and treatment level, including tumor localization techniques, experience of the surgeon, and difficulty of the operation.

In conclusion, we reported the largest TIO cohort to date and the recurrence and nonremission rates following an operation. Nearly nine of 10 patients recovered after one or more surgeries. Female sex, spine tumors, bone tissue–involved tumors, malignancy, and low preoperative serum phosphorus levels were identified as risk factors for refractory outcomes. The significance of using cutoff values of phosphate or FGF23 alone to predict outcomes is limited. And tumor location and expertise of the surgeon are of vital importance for a final cure. We should take them into account when making a treatment decision. Further studies focusing on the refractory mechanisms are of vital importance for TIO treatment.

## Disclosures

All authors state that they have nothing to disclose.

## Supporting information


**Supplemental Table 1** Reasons for non‐remission and recurrence.

**Supplemental Table 2** Sensitivity and specificity of each cut‐off point of preoperative phosphate.

**Supplemental Table 3** Sensitivity and specificity of each cut‐off point of preoperative FGF23.

**Supplemental Table 4** Multivariable regression analysis excluded tumor malignancy.

**Supplemental Table 5** Correlation between onset age and other variables.

**Supplemental Table 6** Regression analyses between FGF23 and outcomes.

**Supplemental Table 7** Comparison of clinical characteristics between non‐remission and recurrent cases.

**Supplemental Fig. 1** Serum phosphate distribution in the refractory and recovery groups. Columns represent the percentage of patients in each interval in the refractory or recovery group (left Y axis). Values on each column indicate the number of cases. The gray curve represents the proportion of refractory cases of all cases in each interval (right Y axis).
