# Supplementary material for: Nonremission and Recurrent Tumor‐Induced Osteomalacia: A Retrospective Study
Source: J Bone Miner Res. 2019 Nov 15;35(3):469–77. doi: 10.1002/jbmr.3903 (PMC7140180; doi:10.1002/jbmr.3903)
Supplement: Supplementary file 1 — Supplemental Table 1 Reasons for non‐remission and recurrence. [file JBMR-35-469-s001.docx]

| **Supplemental Table 1. Reasons for non-remission and recurrence.** | | | | |
| --- | --- | --- | --- | --- |
| Outcomes | Imperfect resection (n = 36) | Distant metastasis (n = 2) | Multiple lesions (n = 2) | Distant metastasis combined multiple lesions (n = 2) |
| Non-remission (n = 24) | 21 | 0 | 2 | 1 |
| Recurrence (n = 18) | 15 | 2 | 0 | 1 |
