# Supplementary material for: Nonremission and Recurrent Tumor‐Induced Osteomalacia: A Retrospective Study
Source: J Bone Miner Res. 2019 Nov 15;35(3):469–77. doi: 10.1002/jbmr.3903 (PMC7140180; doi:10.1002/jbmr.3903)
Supplement: Supplementary file 2 — Supplemental Table 2 Sensitivity and specificity of each cut‐off point of preoperative phosphate. [file JBMR-35-469-s002.docx]

| **Supplemental Table 2. Sensitivity and specificity of each cut-off point of preoperative phosphate** | | | |
| --- | --- | --- | --- |
| **Phosphate (mmol/L)** | **Sensitivity (%)** | **Specificity (%)** | **Youden index** |
| < 0.2100 | 2.381 | 100 | 0.02381 |
| < 0.2350 | 4.762 | 100 | 0.04762 |
| < 0.2500 | 4.762 | 98.4 | 0.03162 |
| < 0.2650 | 4.762 | 97.87 | 0.02632 |
| < 0.2750 | 4.762 | 97.34 | 0.02102 |
| < 0.2850 | 7.143 | 96.81 | 0.03953 |
| < 0.2950 | 7.143 | 95.74 | 0.02883 |
| < 0.3050 | 9.524 | 95.74 | 0.05264 |
| < 0.3150 | 9.524 | 95.21 | 0.04734 |
| < 0.3250 | 14.29 | 95.21 | 0.095 |
| < 0.3350 | 16.67 | 93.09 | 0.0976 |
| < 0.3450 | 21.43 | 90.96 | 0.1239 |
| < 0.3550 | 30.95 | 90.43 | 0.2138 |
| < 0.3650 | 38.1 | 90.43 | 0.2853 |
| < 0.3750 | 40.48 | 89.36 | 0.2984 |
| < 0.3850 | 42.86 | 87.77 | 0.3063 |
| < 0.3950 | 47.62 | 85.64 | 0.3326 |
| < 0.4050 | 50 | 83.51 | 0.3351 |
| < 0.4150 | 50 | 80.85 | 0.3085 |
| < 0.4250 | 50 | 79.26 | 0.2926 |
| < 0.4350 | 54.76 | 77.66 | 0.3242 |
| **< 0.4450** | **61.9** | **73.94** | **0.3584** |
| < 0.4550 | 61.9 | 68.09 | 0.2999 |
| < 0.4650 | 61.9 | 61.7 | 0.236 |
| < 0.4750 | 64.29 | 56.38 | 0.2067 |
| < 0.4850 | 66.67 | 54.79 | 0.2146 |
| < 0.4950 | 66.67 | 51.06 | 0.1773 |
| < 0.5050 | 66.67 | 48.4 | 0.1507 |
| < 0.5150 | 69.05 | 46.81 | 0.1586 |
| < 0.5250 | 73.81 | 44.15 | 0.1796 |
| < 0.5350 | 73.81 | 39.89 | 0.137 |
| < 0.5450 | 73.81 | 36.17 | 0.0998 |
| < 0.5550 | 73.81 | 31.38 | 0.0519 |
| < 0.5650 | 76.19 | 28.19 | 0.0438 |
| < 0.5750 | 78.57 | 23.94 | 0.0251 |
| < 0.5850 | 80.95 | 21.28 | 0.0223 |
| < 0.5950 | 80.95 | 19.15 | 0.001 |
| < 0.6050 | 83.33 | 17.55 | 0.0088 |
| < 0.6150 | 83.33 | 14.89 | -0.0178 |
| < 0.6250 | 83.33 | 12.77 | -0.039 |
| < 0.6350 | 85.71 | 12.23 | -0.0206 |
| < 0.6450 | 88.1 | 11.17 | -0.0073 |
| < 0.6550 | 88.1 | 7.979 | -0.03921 |
| < 0.6650 | 95.24 | 4.787 | 0.00027 |
| < 0.6750 | 95.24 | 3.723 | -0.01037 |
| < 0.6950 | 100 | 2.128 | 0.02128 |
| < 0.7150 | 100 | 1.596 | 0.01596 |
| < 0.7250 | 100 | 1.064 | 0.01064 |
| < 0.7350 | 100 | 0.5319 | 0.005319 |
