# Supplementary material for: Nonremission and Recurrent Tumor‐Induced Osteomalacia: A Retrospective Study
Source: J Bone Miner Res. 2019 Nov 15;35(3):469–77. doi: 10.1002/jbmr.3903 (PMC7140180; doi:10.1002/jbmr.3903)
Supplement: Supplementary file 3 — Supplemental Table 3 Sensitivity and specificity of each cut‐off point of preoperative FGF23. [file JBMR-35-469-s003.docx]

| **Supplemental Table 3. Sensitivity and specificity of each cut-off point of preoperative FGF23** | | | |
| --- | --- | --- | --- |
| **FGF23 (pg/ml)** | **Sensitivity (%)** | **Specificity (%)** | **Youden index** |
| > 48.23 | 100 | 1.538 | 0.01538 |
| > 56.96 | 95.24 | 1.538 | -0.03222 |
| > 65.25 | 95.24 | 3.077 | -0.01683 |
| > 73.81 | 95.24 | 4.615 | -0.00145 |
| > 91.21 | 95.24 | 6.154 | 0.01394 |
| > 106.0 | 95.24 | 7.692 | 0.02932 |
| > 110.3 | 90.48 | 7.692 | -0.01828 |
| > 111.2 | 90.48 | 9.231 | -0.00289 |
| > 119.6 | 90.48 | 10.77 | 0.0125 |
| > 134.5 | 90.48 | 12.31 | 0.0279 |
| > 142.1 | 90.48 | 13.85 | 0.0433 |
| > 154.3 | 90.48 | 15.38 | 0.0586 |
| > 166.1 | 90.48 | 16.92 | 0.074 |
| > 166.6 | 90.48 | 18.46 | 0.0894 |
| > 171.1 | 90.48 | 20 | 0.1048 |
| > 180.8 | 90.48 | 21.54 | 0.1202 |
| > 187.9 | 90.48 | 23.08 | 0.1356 |
| > 190.3 | 90.48 | 24.62 | 0.151 |
| > 195.3 | 85.71 | 24.62 | 0.1033 |
| > 199.7 | 85.71 | 26.15 | 0.1186 |
| > 207.6 | 85.71 | 27.69 | 0.134 |
| > 217.8 | 85.71 | 29.23 | 0.1494 |
| > 231.2 | 85.71 | 30.77 | 0.1648 |
| > 243.9 | 85.71 | 32.31 | 0.1802 |
| > 252.6 | 85.71 | 33.85 | 0.1956 |
| > 259.6 | 85.71 | 35.38 | 0.2109 |
| > 260.3 | 85.71 | 36.92 | 0.2263 |
| > 260.9 | 85.71 | 38.46 | 0.2417 |
| > 267.4 | 85.71 | 40 | 0.2571 |
| > 278.8 | 85.71 | 41.54 | 0.2725 |
| > 286.7 | 85.71 | 43.08 | 0.2879 |
| > 293.6 | 85.71 | 44.62 | 0.3033 |
| > 306.0 | 85.71 | 46.15 | 0.3186 |
| > 317.3 | 85.71 | 47.69 | 0.334 |
| > 322.0 | 85.71 | 49.23 | 0.3494 |
| > 325.0 | 85.71 | 50.77 | 0.3648 |
| > 335.8 | 85.71 | 52.31 | 0.3802 |
| > 355.3 | 85.71 | 53.85 | 0.3956 |
| > 369.9 | 85.71 | 55.38 | 0.4109 |
| > 374.7 | 80.95 | 55.38 | 0.3633 |
| > 375.6 | 80.95 | 56.92 | 0.3787 |
| > 380.1 | 80.95 | 58.46 | 0.3941 |
| > 383.8 | 80.95 | 60 | 0.4095 |
| > 384.2 | 80.95 | 61.54 | 0.4249 |
| > 386.1 | 76.19 | 61.54 | 0.3773 |
| > 396.8 | 71.43 | 61.54 | 0.3297 |
| > 415.9 | 71.43 | 63.08 | 0.3451 |
| > 426.4 | 71.43 | 64.62 | 0.3605 |
| > 427.8 | 71.43 | 66.15 | 0.3758 |
| > 429.4 | 71.43 | 67.69 | 0.3912 |
| > 444.6 | 71.43 | 69.23 | 0.4066 |
| > 464.1 | 71.43 | 70.77 | 0.422 |
| > 478.7 | 71.43 | 72.31 | 0.4374 |
| > 504.8 | 71.43 | 73.85 | 0.4528 |
| > 525.4 | 71.43 | 75.38 | 0.4681 |
| > 545.7 | 66.67 | 75.38 | 0.4205 |
| > 566.7 | 66.67 | 76.92 | 0.4359 |
| > 574.8 | 66.67 | 78.46 | 0.4513 |
| > 588.3 | 66.67 | 80 | 0.4667 |
| > 602.1 | 66.67 | 81.54 | 0.4821 |
| > 612.0 | 66.67 | 83.08 | 0.4975 |
| > 639.3 | 66.67 | 84.62 | 0.5129 |
| **> 709.8** | **66.67** | **86.15** | **0.5282** |
| > 783.0 | 61.9 | 86.15 | 0.4805 |
| > 831.9 | 57.14 | 86.15 | 0.4329 |
| > 868.0 | 57.14 | 87.69 | 0.4483 |
| > 886.0 | 57.14 | 89.23 | 0.4637 |
| > 911.5 | 52.38 | 89.23 | 0.4161 |
| > 979.8 | 52.38 | 90.77 | 0.4315 |
| > 1076 | 52.38 | 92.31 | 0.4469 |
| > 1232 | 52.38 | 93.85 | 0.4623 |
| > 1370 | 47.62 | 93.85 | 0.4147 |
| > 1525 | 42.86 | 93.85 | 0.3671 |
| > 1652 | 38.1 | 93.85 | 0.3195 |
| > 1721 | 38.1 | 95.38 | 0.3348 |
| > 1789 | 33.33 | 95.38 | 0.2871 |
| > 1789 | 33.33 | 96.92 | 0.3025 |
| > 1915 | 23.81 | 96.92 | 0.2073 |
| > 2048 | 14.29 | 96.92 | 0.1121 |
| > 2164 | 4.762 | 96.92 | 0.01682 |
| > 2272 | 4.762 | 100 | 0.04762 |
