# Supplementary material for: Nonremission and Recurrent Tumor‐Induced Osteomalacia: A Retrospective Study
Source: J Bone Miner Res. 2019 Nov 15;35(3):469–77. doi: 10.1002/jbmr.3903 (PMC7140180; doi:10.1002/jbmr.3903)
Supplement: Supplementary file 4 — Supplemental Table 4 Multivariable regression analysis excluded tumor malignancy. [file JBMR-35-469-s004.docx]

| **Supplemental Table 4. Multivariable regression analysis excluded tumor malignancy** | |
| --- | --- |
| **Factor** | OR (95%CI) |
| **Onset age, OR per increase 1 year** | Excluded from equation |
| **Gender** |  |
| Male | 1 |
| Female | 3.28 (1.41, 7.65) ^b^ |
| **Tumor location** |  |
| Head/Neck | 1 |
| Upper extremities | 2.41 (0.15, 39.09) |
| Lower extremities | 3.25 (1.07, 9.92) ^a^ |
| Hip/Pelvic | 3.49 (0.82, 14.84) |
| Spine | 60.54 (7.92, 463.03) ^c^ |
| Other | 2.28 (0.21, 24.25) |
| **Involved tissue** |  |
| Soft tissue | 1 |
| Bone involved | 7.71 (3.09, 19.26) ^c^ |
| **Preoperative serum phosphorus, per increase 0.1 mmol/L** | 0.53 (0.37, 0.78) ^b^ |
| **ALP before operation, per increase 10 U/L** | Excluded from equation |

Abbreviations: OR, odds ratio; CI, confidential interval; ALP, alkaline phosphatase.

^a^ *p* <0.05.

^b^ *p* <0.01.

^c^ *p* <0.001.
