# Supplementary material for: Nonremission and Recurrent Tumor‐Induced Osteomalacia: A Retrospective Study
Source: J Bone Miner Res. 2019 Nov 15;35(3):469–77. doi: 10.1002/jbmr.3903 (PMC7140180; doi:10.1002/jbmr.3903)
Supplement: Supplementary file 5 — Supplemental Table 5 Correlation between onset age and other variables. [file JBMR-35-469-s005.docx]

| **Supplemental Table 5. Correlation between onset age and other variables** | | | | | | |
| --- | --- | --- | --- | --- | --- | --- |
|  | Female gender | Tumor location | Bone tissue | Malignancy | Serum phosphate | Serum ALP |
| Correlation coefficient | 0.148 | -0.006 | -0.167 | -0.252 | 0.175 | -0.242 |
| *p* value | 0.025 | 0.931 | 0.011 | <0.001 | 0.008 | <0.001 |
