# Supplementary material for: Nonremission and Recurrent Tumor‐Induced Osteomalacia: A Retrospective Study
Source: J Bone Miner Res. 2019 Nov 15;35(3):469–77. doi: 10.1002/jbmr.3903 (PMC7140180; doi:10.1002/jbmr.3903)
Supplement: Supplementary file 6 — Supplemental Table 6 Regression analyses between FGF23 and outcomes. [file JBMR-35-469-s006.docx]

| **Supplementary Table 6. Regression analyses between FGF23 and outcomes** | | |
| --- | --- | --- |
| Factor | Univariate | Multivariate |
| FGF23 | 2.484 (1.430, 4.317) ^a^ | 3.278 (1.637, 6.563) ^a^ |

All available FGF23 levels were divided into 4 stratifications according to interquartile range. In multivariate analysis, involved tissue and malignancy were adjusted.

^a^*p* = 0.001
