# Supplementary material for: Nonremission and Recurrent Tumor‐Induced Osteomalacia: A Retrospective Study
Source: J Bone Miner Res. 2019 Nov 15;35(3):469–77. doi: 10.1002/jbmr.3903 (PMC7140180; doi:10.1002/jbmr.3903)
Supplement: Supplementary file 7 — Supplemental Table 7 Comparison of clinical characteristics between non‐remission and recurrent cases. [file JBMR-35-469-s007.docx]

| **Supplemental Table 7. Comparison of clinical characteristics between non-remission and recurrent cases** | | | |
| --- | --- | --- | --- |
| Characteristic | Non-remission | Recurrent | P value |
| Onset age, Mean±SD, years | 34.29±12.74 | 35.11±12.81 | 0.838 |
| Gender, F:M | 10:14 | 7:11 | 0.856 |
| Premenopausal rate in female, % | 100% | 100% | - |
| Duration, median (IQR), years | 4.3 (3.1, 7.8) | 4.0 (2.0, 6.3) | 0.523 |
| Serum phosphate, Mean±SD, mmol/L | 0.43±0.14 | 0.46±0.12 | 0.469 |
| ALP, median (IQR), U/L | 206.5 (137.5, 258.0) | 226.7 (159.3, 309.5) | 0.409 |
| FGF23 before surgery, median (IQR), pg/ml^*^ | 1789.41  (1229.86, 2044.63) | 374.67  (109.57, 387.48) | <0.001 |
| Creatinine, Mean±SD^#^, μmol/L | 57.76±15.56 | 59.17±15.95 | 0.806 |

*Data from 14 non-remission cases and 7 recurrent cases.

^#^Data from 21 non-remission cases and 12 recurrent cases.
