# Supplementary figures and images for: Nonremission and Recurrent Tumor‐Induced Osteomalacia: A Retrospective Study
Source: J Bone Miner Res. 2019 Nov 15;35(3):469–77. doi: 10.1002/jbmr.3903 (PMC7140180; doi:10.1002/jbmr.3903)

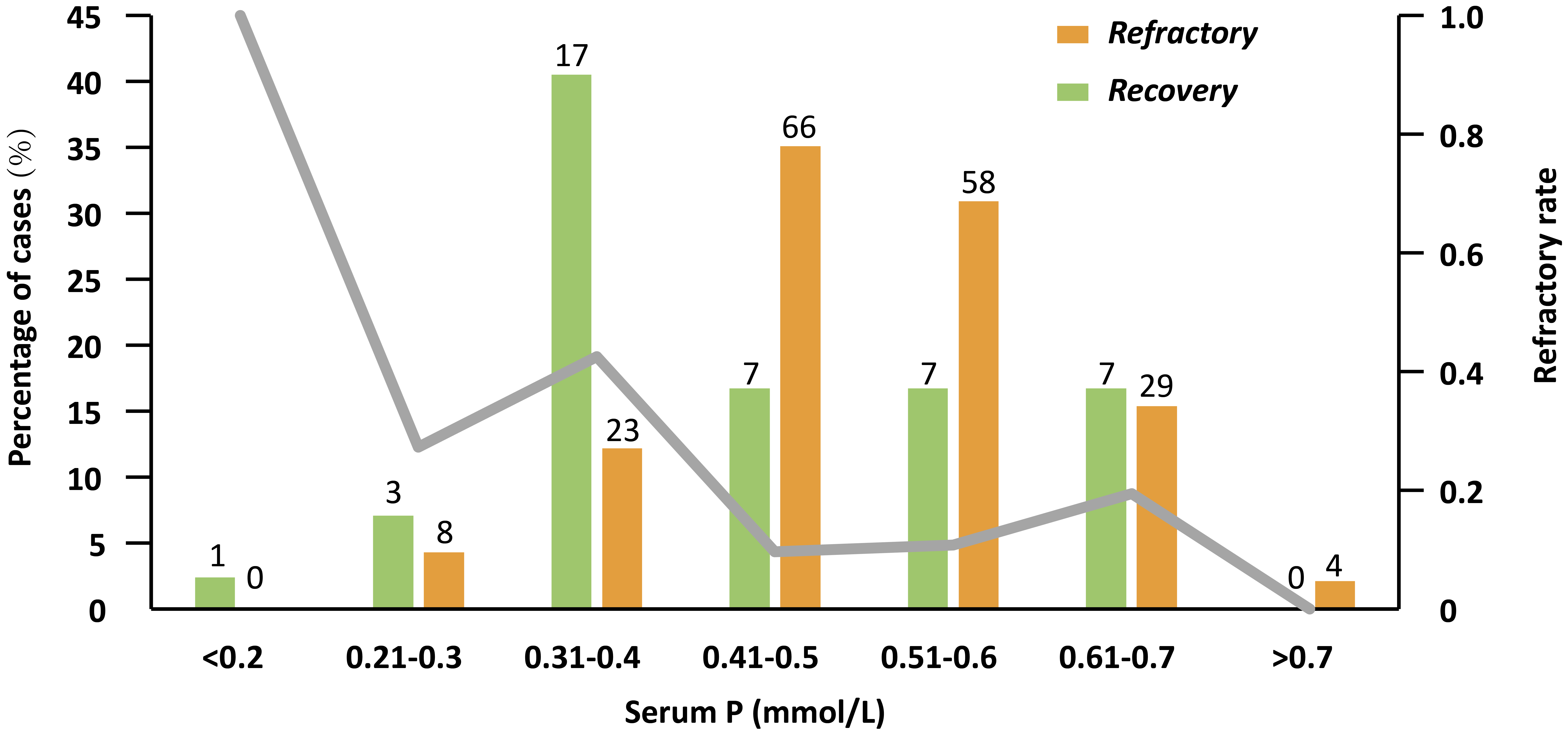

Supplement: Supplementary file 8 — Supplemental Fig. 1 Serum phosphate distribution in the refractory and recovery groups. Columns represent the percentage of patients in each interval in the refractory or recovery group (left Y axis). Values on each column indicate the number of cases. The gray curve represents the proportion of refractory cases of all cases in each interval (right Y axis). [file JBMR-35-469-s008.tif]
